# Implementation of the plasma *MYCN*/*NAGK* ratio to detect *MYCN* amplification in patients with neuroblastoma

**DOI:** 10.1002/1878-0261.12794

**Published:** 2020-09-18

**Authors:** Yan Su, Lijun Wang, Qian Zhao, Zhixia Yue, Wen Zhao, Xisi Wang, Chao Duan, Mei Jin, Dawei Zhang, Shenglan Chen, Jianfeng Yin, Lihua Qiu, Xianfeng Cheng, Zhong Xu, Xiaoli Ma

**Affiliations:** ^1^ Beijing Key Laboratory of Pediatric Hematology Oncology National Discipline of Pediatrics Ministry of Education MOE Key Laboratory of Major Diseases in Children Hematology Oncology Center Beijing Children's Hospital Capital Medical University National Center for Children's Health Beijing China; ^2^ Beijing Keyin Technology Company Limited Beijing Keyin Evergreen Institutes for Medical Research Company Limited China; ^3^ Taizhou Genewill Medical Laboratory Company Limited China

**Keywords:** *MYCN* amplification, *MYCN/NAGK* ratio, neuroblastoma, plasma, qPCR

## Abstract

Detection of amplification of the *MYCN* gene is essential for determining optimal treatment and estimating prognosis of patients with neuroblastoma (NB). DNA FISH with neuroblastoma tissues or patient‐derived bone marrow cells is the standard clinical practice for the detection of *MYCN* amplification. As tumor cells may often be unavailable, we developed a method to detect *MYCN* amplification in the plasma of patients with neuroblastoma. Taking single‐copy *NAGK* DNA as reference, we used real‐time quantitative PCR (qPCR) to determine the *MYCN*/*NAGK* ratio in the plasma of 115 patients diagnosed with NB. An increased *MYCN*/*NAGK* ratio in the plasma was consistent with *MYCN* amplification as assessed by DNA FISH. The AUC for a *MYCN*/*NAGK* ratio equal to 6.965 was 0.943, with 86% sensitivity and 100% specificity. Beyond the threshold of 6.965, the *MYCN*/*NAGK* ratio correlated with a heavier tumor burden. Event‐free and overall survival of two years were significantly shortened in stage 4 patients with a *MYCN*/*NAGK* ratio higher than 6.965. Plasma *MYCN*/*NAGK* ratios increased in patients with progressive disease and relapse. Thus, we conclude that the determination of the plasma *MYCN*/*NAGK* ratio by qPCR is a noninvasive and reproducible method to measure *MYCN* amplification in patients with NB.

## Graphical abstract

Detection of amplification of the *MYCN* gene is essential for estimating treatment and prognosis of patients with neuroblastoma. We developed a method to detect *MYCN* amplification using the plasma DNA by quantitative PCR. An increase in *MYCN* copies in the plasma was consistent with *MYCN* amplification as assessed by DNA‐FISH to measure *MYCN* amplification in patients with neuroblastoma.

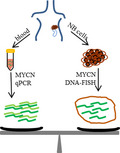

**Abbreviations**
AUC: area under the ROC curve
cfDNA: cell‐free DNA
CI: confidence interval
EFS: event‐free survival
FISH: fluorescence in situ hybridization
LDH: lactate dehydrogenase
NB: neuroblastoma
NSE: neuron‐specific enolase
OS: overall survival
qPCR: quantitative PCR
ROC: receiver operating characteristic

## 1 Introduction

Neuroblastoma (NB) is the most common extracranial solid tumor of pediatric malignancies and originates from sympathetic nervous system, accounting for approximately 10% of all pediatric tumors [1, 2, 3]. Although substantial progresses have been made in therapy of NB, including chemotherapy, radiotherapy, surgery, hematopoietic stem cell transfusion, and immunotherapy, the 5‐year survival rate of patients with high‐risk NB remains still less than 50% [4, 5, 6, 7, 8, 9]. Besides age of onset, primary location, and metastasis, *MYCN* amplification is widely considered to correlate with risk stratification, relapse, progressive disease, and unfavorable survival rate in NB patients [5, 10, 11, 12, 13, 14, 15, 16, 17]. Therefore, the determination of *MYCN* amplification is necessary in patients with NB.

Histological and cytological investigation on tumor tissue, bone marrow, and circulating tumor cells provides significant clinical features, including diagnosis and risk stratification and genetic profile [10, 18, 19, 20]. Presently, fluorescence *in situ* hybridization (FISH) is the most accurate way to evaluate status of *MYCN* amplification of tumor or metastatic bone marrow in NB [5, 10, 12, 17, 21, 22]. However, checking status of *MYCN* amplification is impossible in case that tissue biopsy is unavailable or limited at the time of diagnosis. Bone marrow of metastasis could be selected as alternative sample to examine *MYCN* amplification of bone marrow cells in patients with NB [12, 17, 21, 23]. Unfortunately, negativity of *MYCN* amplification in cells from metastatic bone marrow could not prove *MYCN* nonamplification of tumor because of tumors' heterogeneity [21, 22, 23]. To depict tumor heterogeneity and minimal residual disease, liquid biopsy is recommended to estimate tumor dynamics [24, 25, 26, 27, 28]. Recently, plasma cell‐free DNA (cfDNA) quantification is emerging as a promising and noninvasive method to predict tumor burden in NB [29, 30, 31]. More importantly, serum or plasma *MYCN* copy number quantification by real‐time quantitative polymerase chain reaction (qPCR) was used to predict amplified *MYCN* of tumor in NB, at different cutoff value [32, 33, 34, 35, 36, 37]. Remarkably, less work is done with plasma DNA and more clinical tests are needed. Hereby, the aim of our study was to predict *MYCN* amplification status of tumor using plasma cfDNA‐based qPCR from patients with NB.

## 2 Materials and methods

### 2.1 Patients

A total of 115 patients with NB were recruited at the Hematology Oncology Center, Beijing Children's Hospital between January 1, 2016, and December 31, 2019. The initial diagnosis of NB was made according to International Neuroblastoma Staging System (INSS) criteria. Unequivocal pathologic diagnosis was made from tumor tissue by light microscopy or bone marrow aspirate or trephine biopsy contained unequivocal tumor cells with increased urine/serum catecholamines/metabolites. In special cases of seriously ill patients without bone marrow metastasis, the initial clinical diagnosis was established by typical tumor localization with typical metastases (such as bone, liver, lymph node, and skin) detected by metaiodobenzylguanidine (MIBG) or fluorine‐18‐fluoro‐2‐deoxy‐d‐glucose positron emission tomography/computed tomography (18F‐FDG PET/CT) combined with abnormal tumor marker levels. Patients were staged according to the International Neuroblastoma Risk Group Staging System (INRGSS) and grouped by the INRG classification system. Tumors were classified in accordance with the International Neuroblastoma Pathology Classification System (INPC). All diagnosed patients were followed up by the end of December 2019. This study and the BCH‐NB‐2007 protocol were approved by the Beijing Children's Hospital Institutional Ethics Committee (No. 2016‐65). Informed consent was obtained from the patients' parents or guardians according to the Declaration of Helsinki. The BCH‐NB‐2007 protocol is based on the Hong Kong Pediatric Hematology and Oncology Study Group guidelines [38] and the results of a study in Germany [39]. According to risk stratification of NB, multidisciplinary treatment was applied in HR‐NB patients, including induction chemotherapy, surgery, consolidation therapy, radiotherapy, and autologous stem cell transplantation. The intermediate‐risk patients received chemotherapy and surgery. The patients in low‐risk group received surgery with or without chemotherapy. According to efficacy evaluation of HR‐NB, after the second, fourth, and sixth course of chemotherapy, before stem cell transplantation, before maintenance treatment, every 3 months during maintenance treatment, tumor markers and imaging examination were performed to evaluate the size of tumor focus and metastasis site, and with bone marrow metastasis patients, bone marrow puncture of sternum and ilium were also performed.

### 2.2 Clinical test and evaluation

Upon initial diagnosis, bone marrow biopsies and/or aspirates were obtained for microscopic examination and identification of NB cells. Amplification of the *MYCN* gene was detected by FISH in both resected tumor tissues and bone marrow cells. *MYCN* amplification was defined as a > fourfold increase of *MYCN* signals in relation to the number of chromosome 2 in Fig. S1 as described [22]. Laboratory analysis was performed prior to treatment, and the interval between laboratory tests and biopsy was less than 15 days. Urinary vanillylmandelic acid (VMA) and homovanillic acid (HVA) were analyzed by gas chromatography‐mass spectrometry (GC/MS), and their concentrations were expressed as a ratio to urinary creatinine concentration. Lactate dehydrogenase (LDH) and neuron‐specific enolase (NSE) were measured in serum using routine clinical chemistry laboratory methods. During the period of follow‐up, progressive diseases, relapse, and death were defined as events in patients with NB.

### 2.3 Sample collection

Venous blood samples were collected at the time of diagnosis. Serial blood samples were taken in several patients at 4th cycle of chemotherapy, postsurgery, event occurrence, and ending of follow‐up. Blood samples were collected into ethylenediaminetetraacetic acid‐coated tubes and centrifuged at 1600 ***g*** for 10 min. Supernatants were transferred to fresh tubes and centrifuged at 16 000 ***g*** for 10 min. Plasma was removed and stored at −80 °C until DNA extraction.

### 2.4 Plasma *MYCN*/*NAGK* ratio quantification

DNA was purified from 200 μL of plasma and eluted by 300 μL of elution buffer using QIAamp DNA Blood Mini Kits (Qiagen, Valencia, CA, USA) according to the manufacturer's instructions. Taking *NAGK* (a single‐copy gene) as a reference gene, plasma *MYCN*/*NAGK* ratio was quantified as previously described [33]. SYBR^®^ Green qPCR was performed on a LightCycler LC480 PCR machine (Roche Molecular Systems, Inc., Pleasanton, CA, USA). The sequence of primers of *MYCN* and *NAGK* is as listed as follows:

*MYCN* forward, 5′‐GCAGCAGTTGCTAAAGAA‐3′;
*MYCN* reverse, 5′‐CAGTGACTGTCCAGTTTTG‐3′;
*NAGK* forward, 5′‐TGGGCAGACACATCGTAGCA‐3′;
*NAGK* reverse, 5′‐CACCTTCACTCCCACCTCAAC‐3′.

A serially diluted standardized solution of human genomic DNA (Thermo Fisher Scientific, Waltham, MA, USA) was used to create a reference standard curve. The *MYCN*/*NAGK* ratio was determined by the ratio of the *MYCN* dosage to the *NAGK* dosage according to the standard curve. The qPCRs were performed in triplicate, and mean values of the triplicates were used for further analysis. The qPCR mixture was 10 μL and contained 2 μL of the eluted DNA, 1 μL (final concentration 0.2 μmol) of each forward and reverse primer of *MYCN* or *NAGK*, 5 μL of UltraSYBR Mixture (Cwbiotech, Beijing, China), and 1 μL of double‐distilled water. Cycling conditions were 1 min at 95 °C and 40 cycles of 95 °C for 8 s and 60 °C for 20 s. Each plate contained a plasma DNA sample, a negative control (water template), and seven serially diluted standard DNA solutions (10, 5, 1, 0.5, 0.25, 0.0625, and 0.015 ng·µL^−1^).

### 2.5 Statistics analysis

Data are presented as the median and 95% confidence interval (CI), and were analyzed using the Mann–Whitney *U*‐test or one‐way ANOVA test and chi‐square test in r statistical environment (version 3.4.0, R Foundation for Statistical Computing, Vienna, Austria). Event‐free and overall survival curves were generated by Kaplan–Meier method, and curves were compared using a log‐rank test. Receiver operating characteristic (ROC) curves were constructed and analyzed using the Bioconductor ROC package. A *P*‐value of < 0.05 was considered significant.

## 3 Results

### 3.1 Clinical characters of patients with newly diagnosed NB

Clinical characters of 115 patients are analyzed in Table 1 and Table S1. The male and female patients diagnosed with NB were similar, 51.3% and 48.7%. The median age of diagnosed NB was 36 months, and most NB children ranged from 18 to 60 months. Ninety‐six tumors (83.48%) are primarily found at abdomen. Determined by FISH in NB tumors, amplified *MYCN* was detected in 37 cases, 25 boys and 12 girls. The positive rate of *MYCN* amplification in boys was significantly higher than in girls, 42.37% *vs* 21.43%. More intriguingly, 37 *MYCN* amplification patients were all found in tumors originated at abdomen. This expression pattern provided an evidence that tumor of NB located in abdomen highly correlated to heavier tumor burden and high rate of *MYCN* amplification. In addition, 26 (55.32%) patients with *MYCN* amplification had NSE level more than 370 ng·mL^−1^, and 25 (80.65%) had LDH level more than 1500 IU·L^−1^. For metastatic sites, including bone, bone marrow, lymph node, liver, spleen, and brain, 65 (56.52%) patients had less than three organs involved metastasis, 30 (26.09%) had 3, and 20 (17.39%) had more than 3 metastatic sites, respectively. However, there was no significant difference of *MYCN* amplification between metastatic organ sites.

**Table 1:** Demographic and clinical features of patients with newly diagnosed NB. Amp, amplification; Nonamp, nonamplification; NSE, neuron‐specific enolase; LDH, lactate dehydrogenase; *P*: chi‐square test.

Characters	Total *N* = 115	*N*	%	*MYCN* amp by FISH			*P*
				Amp	Nonamp	% of Amp	
Gender	Male	59	51.30	25	34	42.37	**< 0.05**
	Female	56	48.70	12	44	21.43	
Age (months)	< 18	17	14.78	6	11	35.29	> 0.05
	≥ 18 and ≤ 60	74	64.35	24	50	32.43	
	> 60	24	20.87	7	17	29.17	
Primary site	Abdomen	96	83.48	37	59	38.54	**< 0.05**
	Thorax and other	19	16.52	0	19	0.00	
NSE (ng·mL^−1^)	< 370	68	59.13	11	57	16.18	**< 0.01**
	≥ 370	47	40.87	26	21	55.32	
LDH (IU·L^−1^)	< 500	42	36.52	4	38	9.52	**< 0.01**
	≥ 500 and < 1500	42	36.52	8	34	19.05	
	≥ 1500	31	26.96	25	6	80.65	
Metastasis sites	< 3	65	56.52	15	50	23.08	> 0.05
	3	30	26.09	12	18	40.00	
	> 3	20	17.39	10	10	50.00	

Values in bold highlight the statistical significance with *P* value less than 0.05 or 0.01.

### 3.2 High plasma *MYCN/NAGK* ratio predicting *MYCN* amplification status of tumor

**Fig. 1:**
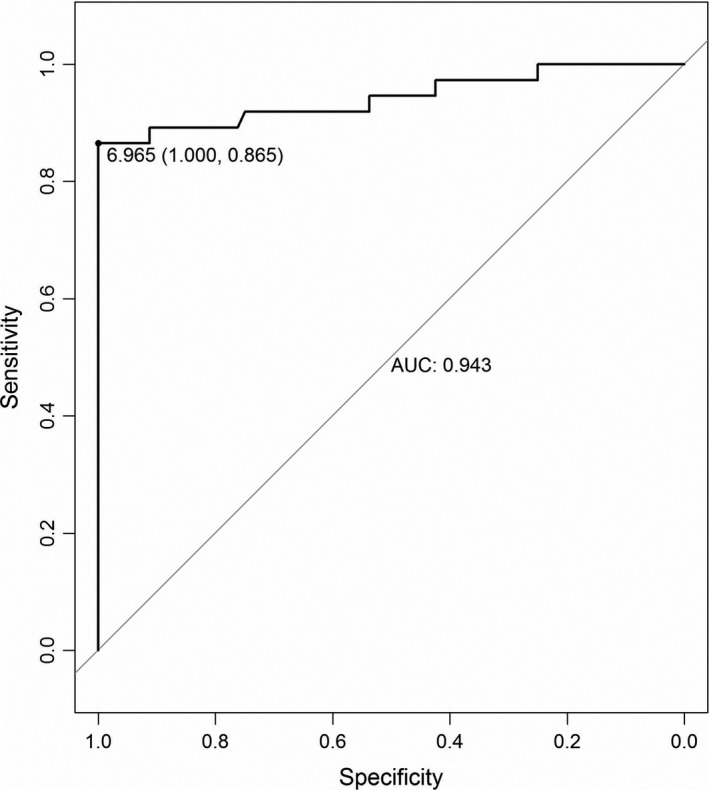
Receiver operating characteristic curve analysis of the predictive value of plasma *MYCN*/*NAGK* ratio for NB at the time of diagnosis. The plasma *MYCN/NAGK* ratio for optimal sensitivity and specificity and the AUC are indicated.

According to the FISH results of tumor *MYCN* status (Table 2, Table S1), plasma *MYCN/NAGK* ratio was significantly higher in *MYCN*‐positive patients with NB than in negative patients, 69.07 (median CI 95%, 53.39, 142.50) *vs* 1.27 (median CI 95%, 1.00, 1.53). It represented plasma *MYCN/NAGK* ratio could discriminate *MYCN* amplification status significantly. To test the performance power of prediction by *MYCN/NAGK* ratio, receiver operating characteristic (ROC) analysis was applied in NB patients. The area under the ROC curve (AUC) was 0.943, with an optimal sensitivity and specificity of 86.5% and 100%, respectively, at a *MYCN/NAGK* ratio of 6.965 (Fig. 1). Other than the powerful performance of *MYCN/NAGK* ratio prediction, higher plasma *MYCN/NAGK* ratio showed consistent with heavier tumor load. For instance, plasma *MYCN/NAGK* ratio was significantly different between primary sites of tumors, serum levels of NSE and LDH, and organ sites of metastasis (Table 2). In particular, plasma *MYCN/NAGK* ratio was significantly higher in NB patients with serum NSE more than 370 ng·mL^−1^ and serum LDH more than 1500 IU·L^−1^ than those with NSE less than 370 ng·mL^−1^ and LDH less than 1500 IU·L^−1^, 26.17 (median CI 95%, 1.71, 60.76) *vs* 1.32 (median CI 95%, 1.00, 1.57), 81.43 (median CI 95%, 40.74, 149.6) *vs* 1.54 (median CI 95%, 1.30, 1.73), or *vs* 1.13 (median CI 95%, 0.89, 1.54), respectively. Patients with metastatic sites more than three had higher *MYCN/NAGK* ratio than those with metastatic sites less than 3, 5.29 (median CI 95%, 1.25, 40.74) *vs* 1.53 (median CI 95%, 1.12, 1.71). Thus, the higher plasma *MYCN/NAGK* ratio indicated heavier tumor burden in NB patients.

**Table 2:** High plasma *MYCN/NAGK* ratio predicting *MYCN* amplification status of tumor.

Character	Subgroup	*N*	Plasma *MYCN* (median, CI 95%)	*P‐*value
FISH‐*MYCN*	Amp	37	69.07 (53.39, 142.50)	**< 0.01** ^a^
	Nonamp	68	1.27 (1.00, 1.53)	
Gender	Male	59	1.73 (1.30,4.53)	> 0.05^a^
	Female	56	1.55 (1.25,1.78)	
Age (months)	< 18	17	1.71 (0.9, 53.39)	> 0.05^b^
	≥ 18 and ≤ 60	74	1.57 (1.37, 2.21)	
	> 60	24	1.59 (0.91, 4.53)	
Primary site	Abdomen	96	1.60 (1.37, 2.63)	> 0.05^a^
	Thorax and other	19	1.56 (0.89, 1.83)	
NSE (ng·mL^−1^)	< 370	68	1.32 (1.00, 1.57)	**< 0.01** ^a^
	≥ 370	47	26.17 (1.71, 60.76)	
LDH (IU·L^−1^)	< 500	42	1.13 (0.89,1.54)	**< 0.01** ^b^
	≥ 500 and < 1500	42	1.54 (1.30,1.73)	
	≥ 1500	31	81.43 (40.74,149.60)	
Metastasis sites	< 3	65	1.53 (1.12, 1.71)	**< 0.05** ^b^
	3	30	2.47 (1.44, 44.26)	
	> 3	20	5.29 (1.25, 40.74)	

^a^ Mann–Whitney *U*‐test.

^b^ Kruskal–Wallis test.

### 3.3 High plasma *MYCN/NAGK* ratio predicting poor outcome of patients with NB in INSS stage 4

**Fig. 2:**
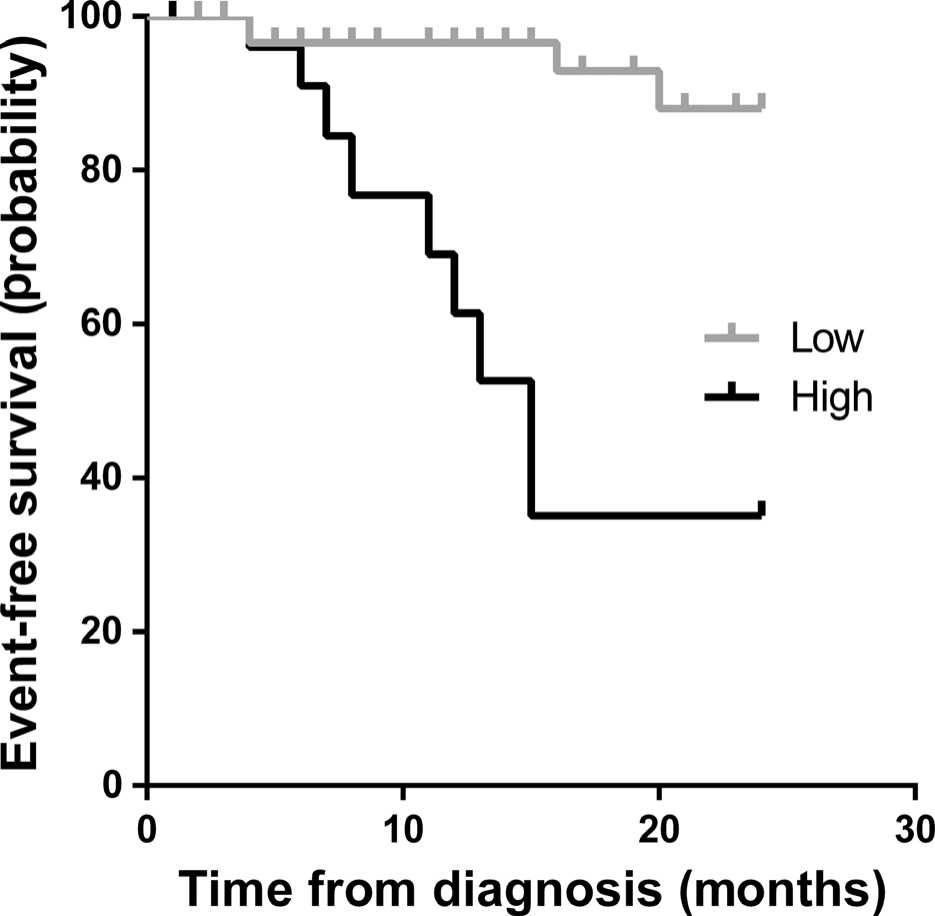
The Kaplan–Meier curve of event‐free survival time in two years. Event‐free curves for patients with plasma *MYCN/NAGK* ratio > 6.965 *vs* those ≤ 6.965.

**Fig. 3:**
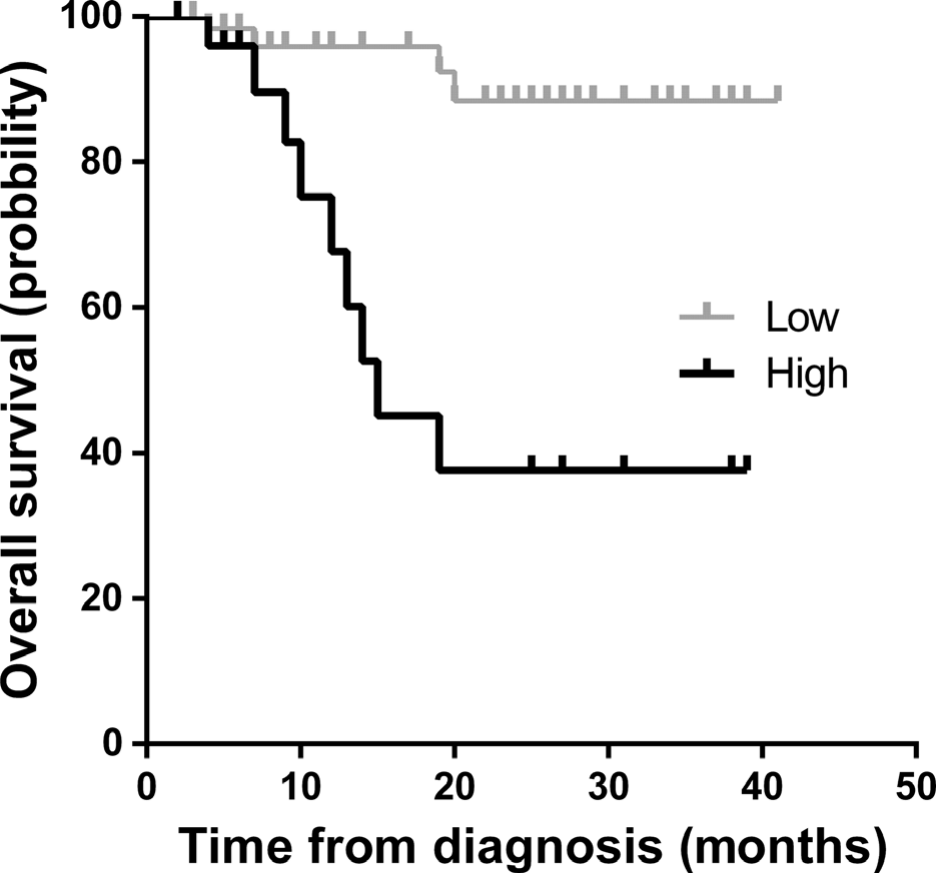
The Kaplan–Meier curve of overall survival time in two years. Overall survival curves for patients with plasma *MYCN/NAGK* ratio > 6.965 *vs* those ≤ 6.965.

Set the threshold of 6.965 according to AUC analysis, event‐free survival time (EFS) and overall survival time (OS) of patients with NB in INSS stage 4 were investigated by Kaplan–Meier curve during 2 years. Events are referred as relapse, progressive disease, and death during follow‐up period. Thirteen in 30 patients (43.33%) appeared to have events in patients with high plasma *MYCN/NAGK* ratio, which was significantly higher than those with low *MYCN/NAGK* ratio 15 in 63 (23.81%) (Table 3). Consistently, EFS was statistically significantly higher for patients with low plasma *MYCN/NAGK* ratio than with high plasma *MYCN/NAGK* ratio (86.61% *vs* 35.10%; Fig. 2). In similarity, the mortality rate was significantly higher in patients with high plasma *MYCN/NAGK* ratio than those with low plasma *MYCN/NAGK* ratio, 33.33% (10 in 30) *vs* 6.35% (4 in 63) (Table 4). Patients with low plasma *MYCN/NAGK* ratio had significantly higher OS than those with high plasma *MYCN/NAGK* ratio (88.41% *vs* 37.59%; Fig. 3).

**Table 3:** Event‐free survival time in patients of stage 4 during two years. *P*: Mantel–Cox test.

*MYCN/NAGK* ratio	*N*	Events	Event‐free	Events %	*P*‐value
High (> 6.965)	30	13	17	43.33	**< 0.05**
Low (≤ 6.965)	63	15	48	23.81	

**Table 4:** Overall survival time in patients of stage 4 during two years. *p*: Mantel–Cox test.

*MYCN/NAGK* ratio	*N*	Mortality	Live	Mortality %	*P*‐value
High (> 6.965)	30	10	20	33.33	**< 0.01**
Low (≤ 6.965)	63	4	59	6.35	

### 3.4 Increased plasma *MYCN/NAGK* ratio indicating therapeutic efficiency and events in patients with NB

**Fig. 4:**
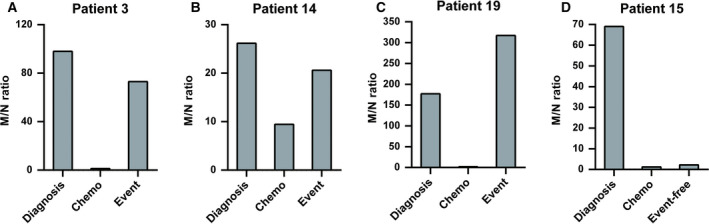
Changes of plasma *MYCN/NAGK* ratio of four patients with *MYCN* amplification during two years. (A–C) patients 3, 14, and 19 with event; (D) patient 15 with remission.

Monitoring therapeutic response and events in patients is necessary and important. Changes of plasma *MYCN/NAGK* ratio were hypothesized to indicate therapeutic response and events happening. In Fig. 4, plasma *MYCN/NAGK* ratio was measured in four patients with *MYCN* amplification at three time points, including diagnosis, adjuvant chemotherapy, event occurrence, and ending of follow‐up. All four patients had high level of plasma *MYCN/NAGK* ratio at diagnosis, ranged from 26.17 to 177.29, far beyond the threshold value 6.965. After four cycles of chemotherapy, plasma MYCN/NAGK ratio declined obviously, less than 10. However, patients' situations were different after multidiscipline treatment. In patient 15 with event‐free by the end of 2‐year follow‐up period, plasma *MYCN/NAGK* ratio was consistently low. In contrast, among patients 3, 14, and 19 with event occurrence, plasma *MYCN/NAGK* ratios raised and were beyond the threshold value 6.965 (in newly diagnosed patients). Remarkably, plasma *MYCN/NAGK* ratio in patient 19 was dramatically higher at the time of event confirmation than at the time of newly diagnosis, 317.37 *vs* 177.29.

## 4 Discussion

Patients with high‐risk NB frequently suffer from low survival rate, less than 50% [7, 9, 19]. Generally, minimal residual disease results in insufficient treatment and recurrence in NB [40, 41, 42]. In clinic, amplification of the *MYCN* is a reliable and powerful indicator both of high‐risk stratification and poor prognosis in NB [11, 12, 17, 43]. Unfortunately, serial assessment of *MYCN* amplification of tumor is not possible due to the lack of primary tumor tissue and heterogeneity of tumor in NB. Nowadays, liquid biopsy test is emerging as a promising and repeatable method to examine tumor load in clinic [24, 26, 44, 45, 46]. Using blood cfDNA from patients with NB sheds light on predicting *MYCN* amplification repeatedly [34, 35, 36]. In our study, the most concern is whether plasma *MYCN/NAGK* ratio could evaluate amplified *MYCN* of NB tumor accurately.

Decade ago, a method‐based real‐time quantitative polymerase chain reaction was developed to measure serum *MYCN*/*NAGK* ratio in NB [33]. When cutoff of serum *MYCN/NAGK* ratio was set at 10, the sensitivity and specificity to distinguish *MYCN* amplification were both 100%. Furthermore, patients with *MYCN/NAGK* ratio beyond 5 had worse overall survival, particularly in those less than 18 months of age [37]. After surgery or neoadjuvant chemotherapy in NB, plasma copy numbers of *MYCN* were significantly decreased [35]. In patients with recurrence or unsuccessful treatment, serum *MYCN/NAGK* ratio was in the higher level than the cutoff value at the time of diagnosis [33]. However, less sensitivity of serum *MYCN/NAGK* ratio in stage 1 or 2 of NB remains improved further [11].

Previous studies demonstrated that plasma cfDNA could be a promising and reproducible method to examine tumor burden of NB [29, 30, 31]. In the present study, plasma *MYCN/NAGK* ratio was tested to predict *MYCN* amplification by qPCR in patients with NB. When threshold was set at 6.965, the performance of plasma *MYCN/NAGK* ratio was 0.943, with 86.5% sensitivity and 100% specificity to discriminate amplified *MYCN* in NB (Fig. 1). The optimal AUC and cutoff value of *MYCN/NAGK* ratios were similar to other investigations by serum [32, 37]. During 2‐year follow‐up, poor prognosis appeared in patients in INSS stage 4 with plasma *MYCN/NAGK* ratio higher than 6.965 (Figs 2 and 3). The powerful prediction of prognosis with plasma *MYCN/NAGK* ratio is consistent to that with FISH examination of *MYCN* amplification in histology [11, 17]. Another advantage of plasma *MYCN/NAGK* ratio to predicting *MYCN* amplification is monitoring therapeutic effect and recurrence disease in NB. Patients with sufficient remission would keep dramatically lower level of plasma *MYCN/NAGK* ratio, while those with progression or recurrence maintain higher level or ascend significantly in NB (Fig. 4). These data are matched with previous finding [33]. It is known that NB tumor originated from abdomen and nonabdomen sites has different outcome and genomic profiles [14, 16]. In comparison with thoracic and neck sites, the 5‐year EFS and OS were lower around 16% and 8% for abdominal primary tumor [16]. Furthermore, abdomen tumors are more likely to harbor *MYCN* amplification than nonabdomen tumors [14, 16]. Consistently, our data show that all 37 tumors with *MYCN* amplification are detected at abdomen exclusively (Table 1).

## 5 Conclusions

In conclusion, plasma *MYCN/NAGK* ratio may be a promising indicator of *MYCN* amplification of tumor in NB. Combined with other clinical features, plasma *MYCN/NAGK* ratio could successfully distinguish heavier tumor burden, insufficient treatment, and poor prognosis. More importantly, plasma *MYCN/NAGK* ratio is a promising, noninvasive, less time‐consuming, and repeatable method to check *MYCN* amplification of tumors in NB when tumor tissues are limited and *MYCN* nonamplification is detected in bone marrow cells by FISH test.

## Conflict of interest

The authors declare no conflict of interest.

## Author contributions

XM and ZX designed the study. XM, YS, and LW conceptualized the study. XM, YS, LW, and QZ revised the manuscript. SC, JY, LQ, XC, and ZX analyzed and interpreted data. ZY, WZ, XW, QZ, CD, MJ, and DZ collected patients' samples. SC, JY, ZY, and LW performed qPCR. WZ, XW, QZ, CD, MJ, and DZ wrote the manuscript. All authors read and approved the final manuscript.

## Supporting information


**Fig. S1.** Typical fluorescence in situ hybridization (*FISH*) images of bone marrow cells from patients with bone marrow metastatic neuroblastoma. (A) The status of normal *MYCN* was detected using a dual‐color probe. Green signals represent the specific probe for *MYCN* (n=2), and red signals stand for centromeric chromosome 2 probes. (B) The status of *MYCN* amplification. *MYCN* signals show more than 50 copies (n=66) within the nuclei and red signals show that there are 3 chromosome 2. (C) The status of *MYCN* amplification. *MYCN* signals show 20‐30 copies (n=26) within the nuclei. Bars=5μm

**Table S1.** All patients' data (supplement). All data of patients are listed, including demographic, diagnosis, staging, pathology, clinical examinations, plasma *MYCN/NAGK* ratios, events and treatment.

## Data Availability

The raw data are available upon reasonable request from the corresponding authors.
